# The Complexity of Modulating Anthocyanin Biosynthesis Pathway by Deficit Irrigation in Table Grapes

**DOI:** 10.3389/fpls.2021.713277

**Published:** 2021-08-18

**Authors:** Maha Afifi, David Obenland, Ashraf El-kereamy

**Affiliations:** ^1^California Table Grape Commission, Fresno, CA, United States; ^2^United States Department of Agriculture, Agricultural Research Service, San Joaquin Valley Agricultural Sciences Center, Parlier, CA, United States; ^3^Department of Botany and Plant Sciences, University of California, Riverside, Riverside, CA, United States

**Keywords:** table grape, anthocyanin, berry color, temperature, antioxidants, hormones, deficit irrigation

## Abstract

Deficit irrigation (DI) is an irrigation scheduling technique that is used in grapes to improve red color development; however, results are not always satisfactory in table grapes. The red color in grapes is mainly due to the plant pigment anthocyanin. In the present study, the anthocyanin biosynthesis in Scarlet Royal grapes (*Vitis vinifera* L.) grown in the San Joaquin and Coachella Valleys, and subjected to two different DI strategies was investigated. The objective of this study was to identify potential regulatory factors that may lead to potential treatments to improve red color in table grapes, especially under warm climate conditions. In both locations, DI induced the expression of several genes involved in three major pathways that control the red color in table grapes: anthocyanin biosynthesis, hormone biosynthesis, and antioxidant system. DI at veraison induced anthocyanin accumulation and enhanced red color in berries at harvest time. However, anthocyanin accumulation was lower at the Coachella Valley compared to the San Joaquin Valley. The lower level of anthocyanin was associated with lower expression of critical genes involved in anthocyanin biosynthesis, such as *flavonoid-3-O-glucosyltransferase* (*UFGT*), myb-related regulatory gene (*R2R3-MYB)* (*MYBA1*), *basic helix-loop-helix* (bHLH) (*MYCA1*) and the *tryptophan-aspartic acid repeat (WDR or WD40) proteins* (*WDR1*). Further, gene expression analysis revealed the association of ABA biosynthesis gene *9-cis-epoxycarotenoid dioxygenase* (*NCED1*), *1-aminocyclopropane-1-carboxylic acid oxidase* (*ACO3*), and the gibberellic acid (GA) catabolic gene *GA2 oxidase* (*GA2ox1*) in the induction of anthocyanin biosynthesis. An increase in the *chalcone synthase* gene (*CHS2*) was observed in response to DI treatments in both sites. However, *CHS2* expression was higher in Coachella Valley after ending the DI treatment, suggesting the involvement of environmental stress in elevating its transcripts. This data was also supported by the lower level of antioxidant gene expression and enzyme activities in the Coachella Valley compared to the San Joaquin Valley. The present data suggested that the lack of grape red coloration could partially be due to the lower level of antioxidant activities resulting in accelerated anthocyanin degradation and impaired anthocyanin biosynthesis. It seems that under challenging warmer conditions, several factors are required to optimize anthocyanin accumulation via DI, including an active antioxidant system, proper light perception, and hormonal balance.

## Introduction

Red color is one of the most critical fruit quality parameters which determines table grape quality and marketability. Even though the red color is a crucial trait for red table grape varieties worldwide, many factors negatively affect this trait causing significant financial loss. Anthocyanin is a pigment responsible for the red color in grapes. Its synthesis occurs through the flavonoid pathway which includes several enzymatic reactions that work together in a multi-enzyme complex to produce the final forms of anthocyanin (He et al., 2010). This pathway is a part of the phenylpropanoid pathway which is regulated by various factors including genetic background, environmental conditions, and cultural practices (Downey et al., 2006; He et al., 2010; Kuhn et al., 2014). In addition to the major function of anthocyanin in grape coloration, interaction of berry phenolic, sugars, acids, aroma compounds and polysaccharides determine the berry and wine organoleptic properties (Garrido-Bañuelos et al., 2021). The anthocyanin pathway has long been a subject of investigation in fruits including grapes (Jaakola, 2013); however, a recent advance in genetics has led to the development of several red table grape varieties that vary in their anthocyanin composition (Costa et al., 2014). In wine grape, differences among grape cultivars and changes during grape ripening affect the extractability of the phenolic compound into the wine (Garrido-Bañuelos et al., 2021).

After anthesis and fruit set, berries grow rapidly due to a rapid level of cell division and elongation to reach a steady state of growth where chemical changes take place. This allows for the berries to start the maturation process, including the synthesis of anthocyanin in the red varieties (Böttcher et al., 2013, 2015). This stage, “veraison,” is characterized by high levels of hormonal changes leading to the induction of some genes involved in the anthocyanin biosynthesis pathway (Boss et al., 1996; Azuma et al., 2007, 2008). As part of the flavonoid pathway, anthocyanin biosynthesis is controlled by several structural genes starting from *chalcone synthase CHS* and ending with *flavonoid-3-O-glucosyltransferase UFGT* (He et al., 2010). Each of the structural genes is controlled by several transcription factors which regulate the function of this gene. For example, *UFGT*, a major and significant gene in anthocyanin biosynthesis, is regulated by the transcription factor *VvMYBA1* (Jeong et al., 2006; Bogs et al., 2007; Azuma et al., 2007). Other transcription factors contribute to the activation of this pathway, it was reported that *basic helix-loop-helix (bHLH)* (*MYCA1*) and the *tryptophan-aspartic acid repeat (WDR or WD40) proteins* (*WDR1*) form a complex to activate the anthocyanin biosynthesis pathway genes in grapes including the *UFGT* (Hichri et al., 2010; Matus et al., 2010; Jiu et al., 2021). Transcription factors bind and activate promoters of the structural genes. This activation is controlled by physiological and environmental conditions (Downey et al., 2006; He et al., 2010).

Anthocyanin biosynthesis is a complex process controlled by several factors. More studies are required to optimize these factors for a specific variety. It is evident that the activation of this pathway is associated with hormones such as abscisic acid (ABA) and ethylene (Coombe and Hale, 1973). At the practical level, ethylene releasing compounds, such as ethephon, are used to induce and improve red coloration in grapes when applied at color break stage (veraison). This treatment increases the internal ethylene evolution and enhances the expression of several genes involved in the anthocyanin biosynthesis pathway (El-Kereamy et al., 2003). Following the same fashion, other chemicals such as ethanol application at veraison showed the same effect in inducing internal ethylene production, which enhances the transcription of the anthocyanin biosynthesis genes and leads to improved anthocyanin accumulation (El-Kereamy et al., 2002). Additionally, an ABA commercial product, ProTone, (Valent, San Ramon, CA) is also used to improve red coloration in table grapes at veraison stage (Balint and Reynolds, 2013; Leão et al., 2015). Despite the success of using ethephon and ProTone in improving red coloration in some table grape varieties, the results in others remain unsatisfactory (Peppi and Fidelibus, 2008).

Some cultural practices showed a significant effect on improving anthocyanin content in grapes, such as deficit irrigation (DI). This technique has become a valuable tool used to improve berry sugar and enhance red color in table as well as wine grapes (Castellarin et al., 2007a, b; Santesteban et al., 2011; Costello and Patterson, 2012; Casassa et al., 2015; Zarrouk et al., 2016). At veraison, DI accelerates the phenylpropanoid pathway and induces genes involved in the anthocyanin biosynthesis, resulting in a high level of coloration (Castellarin et al., 2007a). The mode of action by which DI induced anthocyanin biosynthesis involved the activation of key genes. However, these genes are regulated by other factors such as light, hormones, and other environmental factors (Mazza, 1995). It is suggested that the physiological effect of the deficit in inducing anthocyanin accumulation is related to the induction of ABA, ethylene, sugar, and triggering genes involved in anthocyanin biosynthesis in grapes and other species (Morgan et al., 1990; Chaves et al., 2010; Dubois et al., 2013; Speirs et al., 2013; Rossdeutsch et al., 2016).

Grape responses to hormonal and DI treatments depend on the variety and the environmental conditions (Rossdeutsch et al., 2016). An optimum anthocyanin biosynthesis pathway requires high to moderate temperature during the day and cooler nights. Thus, grape coloration could be impaired when night temperature does not drop to the optimum level to have a reasonable anthocyanin biosynthesis (Mori et al., 2005).

In addition, even though grapevines thrive under Mediterranean warm conditions, high daily temperatures above 35°C negatively affect anthocyanin accumulation (Kliewer, 1977; Tarara et al., 2008; Cohen et al., 2012). It seems that high temperature reduces the level of ABA and negatively affects the expression of the transcription factor *MYBA1* (Yamane et al., 2006), a key transcription factor for the activation of anthocyanin biosynthesis in grapes (Jeong et al., 2006; Azuma et al., 2007; Bogs et al., 2007). Additionally, the poor red coloration of grapes under high temperature condition is well documented (Mori et al., 2004, 2007; Sadras and Moran, 2012; Venios et al., 2020). The physiological effect of high-temperature stress also involved the induction of reactive oxygen species (ROS) which acts as a stress factor inhibiting anthocyanin biosynthesis and promoting its degradation through the activation of enzymes such as peroxidase (POD) (Movahed et al., 2016). Along with hormonal changes during DI, water limitation induced changes in antioxidant enzyme activity. Some of these enzymes being superoxide dismutase (SOD) (Abedi and Pakniyat, 2010; Alscher et al., 2002; Shiriga et al., 2014), POD (Savić et al., 2008), and catalase (CAT) (Ge et al., 2006). It is presumed that the induction of these enzymes protects the plant cell under water limitation conditions.

Even though DI can be used by growers for the same variety, the outcome varies based on the growing regions. Therefore, the objective of the present study is to investigate the anthocyanin biosynthesis in the same table grape variety subjected to two different DI strategies growing in two different climatic regions of California. Also, this study aimed to identify potential plant regulatory factors associated with the induction of several genes known to be involved in anthocyanin biosynthesis, hormone biosynthesis, and antioxidant system that control the accumulation of anthocyanin in table grapes. This study improves our understanding of the complexity of modulating the anthocyanin biosynthesis pathway by DI in table grapes. The study could also help in selecting potential applied treatments that can be tested for improving red color in grapes.

## Materials and Methods

### Site Selection and Treatments

The objectives of this study were being met by using Scarlet Royal (*Vitis vinifera* L.), which is one of the major table grape varieties in California. Scarlet Royal is a red seedless mid-season maturing variety that has large, sweet, firm berries with a neutral flavor (Hashim-Buckey and Ramming, 2008). In this study, grape samples were collected from two DI experiments conducted in 2016 and 2017 at San Joaquin and Coachella Valleys. These two different regions are distinguished in their climate conditions (Table 1). We realize that there is a lot of variability between the two sites, with rootstock being one of the variables. However, we have prior research indicating that there is no significant difference on the marketable yield which is based on color between the Scarlet Royal own-rooted vines and those grafted on Freedom rootstock (El-Kereamy and Fidelibus, 2018). The focus of this study is to investigate the modulation of anthocyanin pathway by DI regardless of the reasons behind this variation. We used the two locations as examples of upregulated and downregulated anthocyanin pathway and linked the obtained results to other factors including hormones and antioxidant enzymes activity. The goal of this study is to identify some factors that can be used in the field to improve anthocyanin biosynthesis and then improve red color in table grapes.

**TABLE 1:** Monthly climate data reported by CIMIS system (https://cimis.water.ca.gov) in both San Joaquin and Coachella Valleys in the 2016 and 2017 growing seasons.

Region	San Joaquin Valley											
Year	2016						2017					
Month	May	June	July	August	September	October	May	June	July	August	September	October
Total ETo (mm)	185.91	213.63	222.14	198.1	150.34	87.39	176.18	202.89	213.97	188.57	142.48	97.32
Total precip (mm)	8.6	0	0	0	0	9.4	0.1	0	0	0	0.7	0
Avg sol rad (W/sq.m)	308	344	338	306	253	167	294	337	325	286	243	190
Avg vap pres (kPa)	1.2	1.2	1.3	1.3	1.1	1.2	1.2	1.5	1.6	1.6	1.4	0.9
Avg Max air temp (C)	29.1	34.8	36.5	35.9	32.4	25.8	28.1	33.4	36.6	36.1	31.4	25.5
Avg min air temp (C)	13.4	16.7	17.8	16.5	13.1	9.9	12.5	16.4	18.5	17	12.9	6.3
Avg air temp (C)	21.1	26	27.5	26.4	22.8	17.4	20.6	25.4	28.1	27.4	22.6	15.4
Avg max rel hum (%)	77	68	66	72	75	84	75	75	75	83	82	87
Avg min rel hum (%)	26	19	17	18	20	35	29	26	21	22	26	27
Avg rel hum (%)	47	36	35	38	41	59	48	45	41	44	49	53
Avg dew point (C)	8.9	9.7	10.3	10.8	8.7	9.3	8.8	11.9	13.4	14.1	11.4	5.9
Avg wind speed (m/s)	1.8	1.6	1.5	1.4	1.4	1.3	1.7	1.5	1.3	1.4	1.4	1.1
Avg soil temp (C)	25.4	30.2	29.8	28.4	24.4	20.3	25.3	29.7	31.7	29	25.1	18.4

**Region**	**Coachella Valley**											
**Year**	**2016**						**2017**					
**Month**	**May**	**June**	**July**	**August**	**September**	**October**	**May**	**June**	**July**	**August**	**September**	**October**

Total ETo (mm)	210.08	218.46	230.45	213.41	155.75	119.25	214.82	234.63	224.38	209.3	156.17	130.86
Total precip (mm)	0	0	0	0	0	0	0	0	0	0	0	0
Avg sol rad (W/sq.m)	318	320	309	278	225	196	314	345	299	281	240	210
Avg vap pres (kPa)	1.2	1.6	1.7	1.7	1.4	1.3	1	1.3	1.9	1.8	1.6	1
Avg max air temp (C)	32.3	39.9	41.1	40.3	36.1	32.2	34.1	40	41.1	40.7	35.8	33.1
Avg min air temp (C)	15.7	21.2	23.4	24.6	19.3	16.7	16	22	26.3	25.7	20.9	15.6
Avg air temp (C)	24.2	30.8	33	33.1	27.8	24.1	25.6	31.1	33.7	33	27.9	22.9
Avg max rel hum (%)	73	73	66	61	70	68	58	59	59	63	71	65
Avg min rel hum (%)	18	16	17	20	20	24	15	13	20	19	24	18
Avg rel hum (%)	38	37	34	35	38	43	32	29	36	35	45	35
Avg dew point (C)	8.8	13.2	14.6	15	11.5	9.9	6.5	10.3	16.4	15.4	14.4	7
Avg wind speed (m/s)	2.2	2	1.9	1.8	1.8	1.6	2.2	2	1.8	1.8	1.6	1.5
Avg soil temp (C)	23.5	28.3	29.5	29.7	26.4	23.3	23.7	27.7	31.2	29.4	26.8	21.3

### San Joaquin Valley Site and Treatments

At this site, vines were mature (7 years old), grafted on Freedom rootstock, and grown in sandy soil. Spacing at this site was 1.83 × 3.66 meter and the average number of clusters was 35 per vine. The different irrigation treatments used in this site were based on the actual crop water used (ETc) estimated by the Surface Renewal system (SR) (Tule Technologies Inc., CA, United States). Treatments started from the beginning of the season (late-March), and the DI treatments started when grapes were at mid-veraison (mid-July). Treatments were as the following: (1) beyond 120% ETc using existing methods based on soil water measurements with 3 weeks of 75% ETc deficit treatment at mid-veraison (no DI); (2) irrigation hours as recommended by Tule technologies for the entire season without any deficit period leading to 120% ETc (low DI); (3) 100% ETc as estimated by Tule with 3 weeks of 50% ETc deficit treatment at mid-veraison (moderate DI), and (4) a sustained treatment of 80% of ETc as estimated by Tule with 3 weeks of 50% of ETc deficit treatment at mid-veraison (high DI) (Supplementary Figure 1A). Data collection in the present study included three-time sampling. The first sample (before DI) was collected 1 day before applying the deficit irrigation treatment at mid-verasion. The second sample (during DI) was collected 2 days after applying the DI at mid-verasion. The third sample (after DI) was collected 2 days after ending the deficit treatment and re-irrigating vines with the original schedule treatments. The vine water status, as characterized by the leaf water potential (LWP) was measured using 600D Pressure Chamber Instrument (PMS Instrument Company, OR, United States). In both seasons, data of the LWP before DI, during DI, and after re-irrigating vines with the original schedule confirmed that vines responded to the different treatments (Supplementary Figure 2).

### Coachella Valley Site and Treatments

At this site, vines were mature (7 years old), own-rooted and grown in loamy, sandy soil. Spacing at this site was 1.83 × 3.66 meter and the average number of clusters was 32 per vine. DI treatments in this site consisted of complete irrigation cut-off, starting from 2 weeks prior to verasion and lasting for different time durations. Treatments were: (1) full irrigation for the entire season (no DI); (2) full irrigation until veraison (10% softening), followed by no irrigation for 18 in 2016 and 21 days in 2017 (low DI); (3) full irrigation until veraison, followed by no irrigation for 28 days in 2016 and 35 days in 2017 (moderate DI) and (4) full irrigation until veraison, followed by no irrigation for 38 in 2016 and 49 days in 2017 (high DI) (Supplementary Figure 1B). The timing of ending the cut-off irrigation period and starting the re-irrigation in both years was based on three parameters: soil matric potential dropped below −60 centibars at both one and three foot depth; leaf water potential approached −14 bars and trunk maximum daily shrinkage (MDS) measured by dendrometer (Dynamax, CA, United States) approached 100 microns. Samples were collected at three different times. The first sample (before DI) was 1 day before cut-off the irrigation (early in May, at veraison). The second sample (during DI) was collected 15 days after cut-off of the irrigation (mid-May, at veraison). The third sample (after DI) was collected 1 week after re-irrigating the most severe deficit treatment (high DI) (early in June). The vine water status, as characterized by the leaf water potential was measured as mentioned above. In both seasons, data of the LWP before DI, during DI, and after re-irrigating vines with the original schedule confirmed that vines responded to the different treatments (Supplementary Figure 2).

In both sites, at each sampling time, fifty berries were collected from the mid-section of well-exposed south facing clusters for each treatment. To ensure that sampling did not reduce the crop load or affect berry biochemical composition, samples were collected from different vines at each time. Immediately after sampling, the skin of the berries was peeled off, frozen immediately using liquid nitrogen, transferred to the laboratory using dry ice, and stored at −80°C. Frozen samples were then used for further biochemical and molecular purposes.

### Berry Color Determination and Fruit Quality Analysis

**FIGURE 1:**
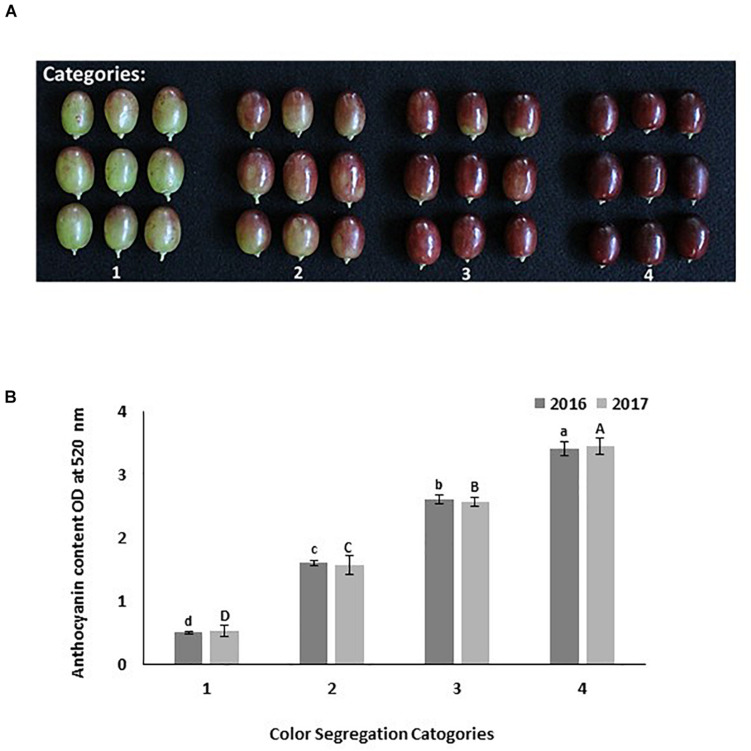
Different categories used to evaluate Scarlet Royal grapes color visually at harvest time. Categories are: 1 = 25% red color; 2 = 50% red color; 3 = 75% color (bright red colored berries with green shoulder); 4 = 100% color (dark red colored berries with no green shoulder) **(A)**. The total anthocyanin content in each color category as measured spectrophotometrically in 2016 and 2017 **(B)**. The error bars represent the standard deviation. Different letters on bars indicate significant differences among categories within the same year at *p* < 0.05 according to the Tukey HSD test within the same season. Different lower-case letters indicate a significant difference among categories in 2016 while capital letters are used for 2017.

In both sites at harvest time, fifty berries from each treatment were collected randomly from clusters located in the inner, central, and outer portion of the canopy. These berries were used to determine the color visually by using the color segregation method (Figure 1A). Berries were separated into four categories: 1 = 25% color; 2 = 50% color; 3 = 75% color with green shoulders, and 4 = 100% color without green shoulders. The color of each treatment was determined by identifying the category under which 50% of berries or more were found. Then, the skin of these berries was removed and frozen immediately to extract and determine the total berry’s skin anthocyanin content. The total anthocyanin content in each color category was quantified to confirm the visual differentiation in color (Figure 1B). Another set of fifty berries per replicate were collected to determine berry weight, length, and diameter. Then these berries were macerated in an electric blender, filtered through a paper towel, and an aliquot of juice was used to determine soluble solids (Brix), pH, and titratable acidity (TA). Soluble solids were determined using a digital temperature compensated refractometer (Milwaukee MA871, AZ, United States). The TA and pH were determined by titrating a 40 mL aliquot of juice with 0.1 N NaOH to a pH of 8.2 using an automatic titrator Excellence T5 (Mettler-Toledo, OH, United States). Fruit quality measurements are presented in Supplementary Table 1.

### Total Anthocyanin Extraction and HPLC Analysis

The frozen skin samples collected at harvest were ground using a ball mill MM300 (Retch) in the presence of liquid nitrogen. One gram of frozen ground samples was extracted into 5 mL of methanol: HCl (100:1). This extraction was used to determine total anthocyanin content spectrophotometry at 520 nm as described by Boss et al. (1996). Total anthocyanin extract was also used to determine anthocyanin composition using high-performance liquid chromatography (HPLC) as described by Downey and Rochfort (2008). HPLC separation of anthocyanins was utilized by using 10% formic acid in water (Solvent A) with a 10% formic acid in methanol (Solvent B) gradient at a flow rate of 1.0 mL/min as described by Downey and Rochfort (2008). The column temperature was maintained at 40°C for the duration of the analysis. The column selected was a Zorbax SB-C18 (150 mm × 4.6 mm, 5μ; Agilent, Palo Alto, CA) protected by an Agilent C-18 guard column. Samples were run on an Agilent 1200 HPLC system equipped with a diode array detector. The gradient conditions were: 0 min, 18% B; 14 min, 29% B; 16 min, 32% B; 18 min, 41% B; 18.1 min, 30% B; 29 min, 41% B; 32min, 50% B; 34.5 min, 100% B; 35–38 min, 18% B. Anthocyanins were monitored by diode array detection at 520 nm. A tentative identification was made by comparison of the spectra and elution times with previously published results and with three purchased standards (malvidin-3-O-glucoside, cyanidin-3-O-glucoside, and petunidin-3-O glucoside) from Alkemist Labs, Costa Mesa, CA. Quantification was performed using calibration curves of the available standards. We determined the berry skin dry weight in the different treatments, and we did not observe any significant difference among treatments thus, HPLC data is expressed on a fresh weight basis. For those compounds in which no standard was available, estimates of concentration were made by utilizing the standard curve for malvidin-3-O-glucoside.

### Quantitative Real Time PCR (RT-qPCR)

Total RNA was isolated from berry skin samples collected during and after DI treatments per the protocol described by Boss et al. (1996). DNA was removed from samples using RNase-free RQI treatment per the manufacturer’s instructions (Promega, Madison, WI, United States) followed by a cleanup with the RNeasy mini kit (Qiagen, Valencia, CA, United States). cDNA was synthesized from total RNA using the qScript^TM^ cDNA Synthesis kit (Quanta Biosciences, MD, United States). Quantitative real-time expression was performed using PerfeCTa SYBR Green SuperMix ROX (Quanta Biosciences, MD, United States) on the ABI7300 real-time system (Applied Biosystems, CA, United States). RT-qPCR was performed using specific primers for the following genes: *flavanone 3-hydroxylase* (*F3H*), *chalcone synthase* (*CHS2*), *myb-related regulatory gene (R2R3-MYB)* (*MYBA1*), *UDP-glucose: flavonoid-3-O-glucosyltransferase* (*UFGT*), *9-cis-epoxycarotenoid dioxygenase* (*NCED1*), *superoxide dismutase1* (*SOD1*), *superoxide dismutase3* (*SOD3), ascorbate peroxidase1 (ASPX1*), *ascorbate peroxidase3 (ASPX3*), *gibberellic acid (GA) catabolic gene GA2 oxidase* (*GA2ox1*), *1-aminocyclopropane-1-carboxylic acid oxidase (ACO3*), *basic helix-loop-helix (bHLH)* (*MYCA1) and tryptophan-aspartic acid repeat (WDR or WD40) protein* (*WDR1*). Primers were designed using the Primer Express 2.0 software (Applied Biosystems) and presented in Supplementary Table 2. Relative quantification for each target gene was calculated by the 2^–ΔΔ^ ^CT^ method (Livak and Schmittgen, 2001) using the grape ubiquitin gene as a constitutive control (Fujita et al., 2005). Ubiquitin primers were designed from a tentative consensus sequence TC38636 and used as constitutive control. RT-qPCR analyses were performed using three biological replicates using sets of cDNA from independent samples. Gene expression during and after DI was calculated as a fold change in reference to the first sampling date (before DI) for each individual site.

### Antioxidant Enzyme Assay

Frozen 0.5 g of berry skin collected before, during and after DI treatments was extracted in 5 mL of 50 mM sodium phosphate buffer (pH 7.0) containing 1 mM ethylenediaminetetraacetic acid (EDTA) and 5% soluble polyvinyl pyrrolidone (Sigma-Aldrich, St. Louis, MO, United States). The resulting homogenate was centrifuged at 3,000 g for 30 min at 4°C and the supernatant was utilized for subsequent measurement of polyphenol oxidase (PPO), peroxidase (POD), and superoxide dismutase (SOD) activities.

The activity of PPO was determined as described by De Pieri Troiani et al. (2003). The standard reaction mixture consisted of 3.4 mL of 100 mM sodium phosphate buffer (pH 6.0); 0.4 mL of catechol (Sigma, NY, United States); 0.2 mL of enzyme extract and the absorbance at 420 nm was measured after 10 min. An enzymatic activity unit was defined as the increase of an absorbance change unit per minute per gram fresh skin weight.

The activity of POD was assayed as described by Troiani et al. (2003), one hundred microliter aliquot of tissue extracts was added to 3 mL of assay solution, consisting of 13 mM guaiacol (Amresco, OH, United States), 5 mM H_2_O_2_, and 50 mM sodium phosphate buffer (pH 7.0) (Cheng et al., 2013). Increases in optical density were then measured at 470 nm for 3 min at 25°C. POD activity was expressed as the change in absorbance per minute per gram fresh skin weight.

Total SOD activity was assayed by monitoring the inhibition of the photochemical reduction of nitro blue tetrazolium (NBT; Roch, United States), which produces a blue coloration in the presence of reactive oxygen, using a Multiskan Go UV-Vis Spectrophotometer (Thermo Scientific, MA, United States) as described previously by Cheng et al. (2013). In brief, 3 mL reaction mixtures comprising 50 mM sodium phosphate buffer (pH 7.0), 13 mM methionine, 75 μM NBT, 2 μM riboflavin, 0.1 mM EDTA (Sigma-Aldrich, St. Louis, MO, United States), and one hundred microliters enzyme extract were illuminated for 20 min at an intensity of 4000 lux using a white fluorescence lamp and then assayed at 560 nm. One unit of SOD activity was defined as the amount of enzyme required to cause 50% inhibition of NBT reduction at 560 nm.

### Experimental Design and Statistical Analysis

At both sites, there were four irrigation treatments with four replications. Irrigation treatments were in a randomized complete block design for both seasons. Two-way ANOVA Statistical analyses were performed using SigmaStat (Systat, CA, United States). The significant difference between treatments and locations was evaluated within the same season using Tukey’s Honestly Significant Difference (HSD) multiple comparison test (*p* ≤ 0.05).

## Results

### Color Segregation at Harvest

**FIGURE 2:**
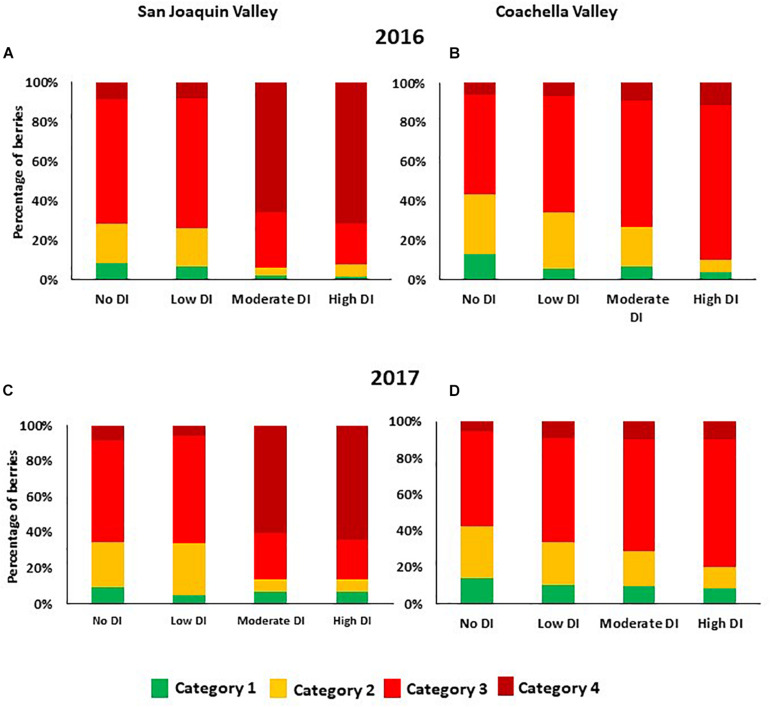
Percentage of berries that fall within the different color categories as affected by deficit irrigation treatments in the 2016 and 2017 seasons in San Joaquin Valley **(A,C)**, and Coachella Valley **(B,D)**. Data represent the average segregation of 200 berries collected from each treatment at harvest time. The different color categories are: 1 = 25% red color; 2 = 50% red color; 3 = 75% color (bright red colored berries with green shoulders); 4 = 100% color (dark red colored berries with no green shoulders).

Under San Joaquin Valley conditions, moderate and high DI treatments enhanced berry color in both years. In these two treatments, more than 60% of berries were found under the 4th category (100% dark red with no green shoulders). However, in the other two treatments which received a high amount of water during the season, no and low DI treatments, 57% or more of berries were found under the 3rd category (75% bright red with green shoulders) (Figures 2A,C). Interestingly, in 2016 and 2017 data showed that in the Coachella Valley, 50% or more berries from the different irrigation treatments were found under the 3rd category (75% color with green shoulders) (Figures 2B,D). Further, in this location a small percentage of berries were found under the 4th category (100% red color without green shoulders) in all DI treatments (Figures 2B,D). Data also illustrated that as the severity of DI increased the percentage of berries under the 2nd category decreased, and the percentage of berries under the 3rd and 4th categories increased. This data suggested that under the Coachella Valley conditions, cutting off the irrigation for longer-term (high DI) enhanced berry color.

### Total Anthocyanin Content

**FIGURE 3:**
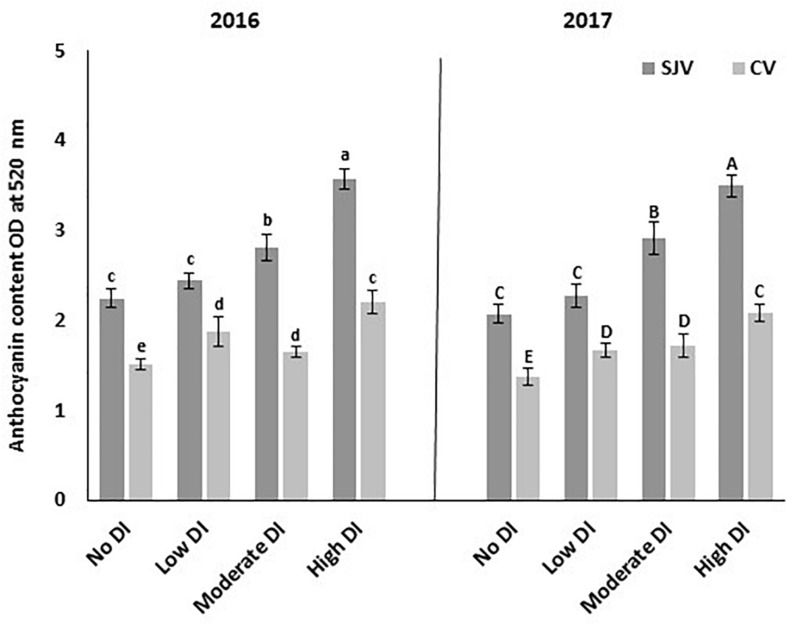
Total anthocyanin contents in Scarlet Royal berry skin at harvest in 2016 and 2017, as affected by different deficit irrigation treatments in San Joaquin Valley (SJV), and Coachella Valley (CV). The data is presented as an optical density (OD) at 520 nm. The error bars represent the standard deviation. Different letters on bars indicate significant differences among treatments and locations at *p* < 0.05 according to the Tukey HSD test within the same season. Different lower-case letters indicate a significant difference among treatments and locations in 2016 while capital letters are used for 2017.

The total berry skin anthocyanin contents of the different treatments at harvest for the 2016 and 2017 seasons are presented in Figure 3. Data showed that in San Joaquin Valley, as the DI severity increased, the total berry skin anthocyanin contents increased (Figure 3). However, no significant differences in the total berry skin anthocyanin were detected between the low DI treatment compared to the fully irrigated treatment (no DI). In both years, the total anthocyanin content of the high DI was significantly higher compared to the other treatments, which received more amount of water during the season.

In Coachella Valley, all DI treatments increased total anthocyanin contents significantly compared to the fully irrigated treatment (no DI) (Figure 3). However, the highest total anthocyanin content was observed in the high DI treatment. Further, no significant differences in the total anthocyanin contents were found between the low and moderate DI treatments (Figure 3).

Generally, total anthocyanin contents in Coachella Valley were lower than in San Joaquin Valley for the different irrigation treatments. Although the vines in Coachella Valley were exposed to high DI increased anthocyanin content significantly; this increment was still at the level of the values obtained in the well-watered treatment (no DI) in San Joaquin Valley in both years.

### HPLC Analysis for Anthocyanin Compounds

**FIGURE 4:**
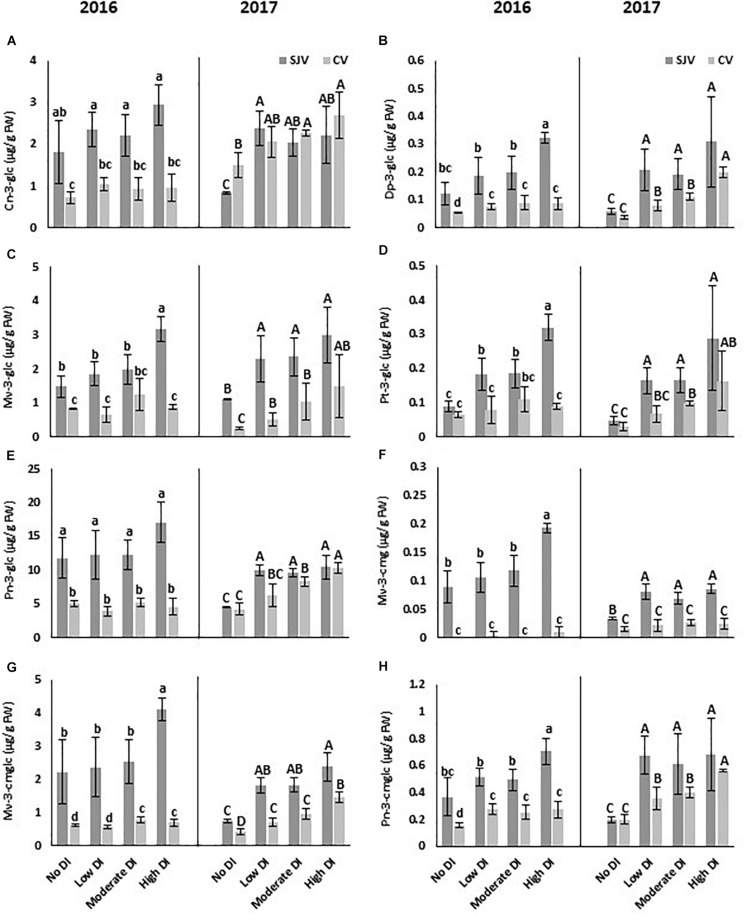
HPLC analysis of different anthocyanin compounds in Scarlet Royal berry skin at harvest in 2016 and 2017, as affected by different deficit irrigation treatments in San Joaquin (SJV) and Coachella (CV) Valleys. The compounds are: cyanidin-3-O-glucoside (Cn-3-glc) **(A)**, delphinidin-3-glucoside (Dp-3-glc) **(B)**, malvidin-3-O-glucoside (Mv-3-glc) **(C)**, petunidin-3-O-glucoside (Pt-3-glc) **(D)**, peonidin-3-O-glucoside (Pn-3-glc) **(E)**, malvidin-3-(6-O-coumaroyl)glucoside (cis) (Mv-3-cmg) **(F)**, malvidin-3-(6-O-coumaroyl)glucoside (trans) (Mv-3-cmglc) **(G)**, and peonidin-3-(6-O-coumaroyl)glucoside (Pn-3-cmglc) **(H)**. The error bars represent the standard deviation. Different letters on bars indicate significant differences among treatments and locations at *p* < 0.05 according to the Tukey HSD test within the same season. Different lower-case letters indicate a significant difference among treatments and locations in 2016 while capital letters are used for 2017.

At harvest of 2016 and 2017 seasons, eight anthocyanin compounds in both sites were quantified by HPLC (Figure 4). The compounds were: cyanidin-3-O-glucoside (Cn-3-glc), delphinidin-3-glucoside (Dp-3-glc), malvidin-3-O-glucoside (Mv-3-glc), petunidin-3-O-glucoside (Pt-3-glc), peonidin-3-O-glucoside (Pn-3-glc), malvidin-3-(6-O-coumaroyl)glucoside (cis) (Mv-3-cmg), malvidin-3-(6-O-coumaroyl)glucoside (trans) (Mv-3-cmglc), and peonidin-3-(6-O-coumaroyl)glucoside (Pn-3-cmglc). Data obtained by HPLC indicated that the major anthocyanin compound in the Scarlet Royal grapes in all samples collected from both sites in 2016 and 2017 was Pn-3-glc, which had the greatest concentration of all compounds (Figure 4E).

Generally, deficit irrigation treatments increased the anthocyanin compounds content significantly compared to the fully irrigated treatment (no DI) (Figure 4). This data supported the data obtained spectrophotometrically for the total anthocyanin. In both sites, data collected in 2016 and 2017 revealed that various anthocyanin compounds followed a different trend. For example, Cn-3-glc was induced in 2017 by DI treatments in both sites, however, no significant difference was observed among treatments in 2016 (Figure 4A). Data also indicated that, although, in 2017 all DI treatments caused a significant increase in Cn-3-glc in San Joaquin Valley, only the moderate and high DI treatments increased it significantly compared to the well-watered treatment in Coachella Valley (Figure 4A). Dp-3-glc showed a significant increase following all DI treatments in both locations during both years (Figure 4B). However, the highest increase was observed following the high DI. Mv-3-glc increased significantly in all DI treatments in both locations in 2017, however, in 2016 it was induced only by the high DI treatment in San Joaquin Valley (Figure 4C). In San Joaquin Valley, Pt-3-glc increased in all DI treatments in both seasons (Figure 4D). However, in Coachella Valley, the induction was observed only in 2017 in the moderate and high DI treatments compared to the other treatments (Figure 4D). Pn-3-glc did not show a significant change in both locations during 2016 season (Figure 4E). In 2017, although, all DI treatments induced the accumulation of Pn-3-glc in San Joaquin Valley, only the moderate and high DI treatments increased in the Coachella Valley (Figure 4E). Data also indicated that, although, in 2017 all DI treatments caused a significant increase in Mv-3-cmg in San Joaquin Valley, only the high DI treatment increased it significantly in 2016 (Figure 4F). Further, in Coachella Valley, no significant difference was observed in Mv-3-cmg accumulation among DI treatments in both years. Moreover, in San Joaquin Valley, Mv-3-cmglc was induced by the high DI treatment in 2016, however, all DI treatments increased the accumulation of this compound in 2017 (Figure 4G). In Coachella Valley, although, Mv-3-cmglc content in 2016 increased significantly by the moderate and high DI treatments, it was induced by all DI treatments in 2017. Further, in 2017, the maximum increase of this compound in the Coachella Valley was observed when applying the high DI treatment (Figure 4G). Also, in San Joaquin Valley, Pn-3-cmglc increased significantly in 2016 by applying the high DI treatment, however, it induced by all DI treatments in 2017 compared to the well-watered vines (Figure 4H). Further, in Coachella Valley, Pn-3-cmglc was increased significantly by DI treatments in both years. However, in 2017, Pn-3-cmglc content was significantly higher in the high DI treatment compared to the other treatments (Figure 4H).

### Gene Expression Analysis of Anthocyanin Biosynthesis Pathway

**FIGURE 5:**
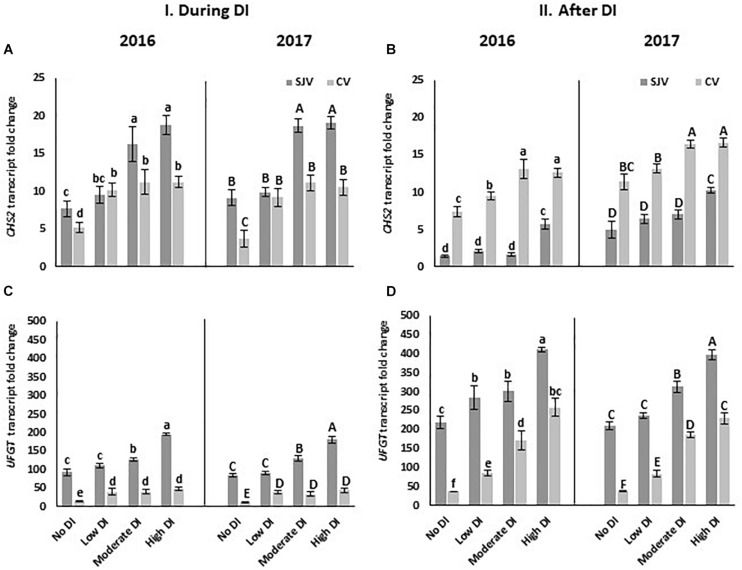
Expression of genes involved in anthocyanin biosynthesis pathway in Scarlet Royal berry skin as affected by deficit irrigation (DI) treatments in 2016 and 2017, in San Joaquin (SJV) and Coachella (CV) Valleys. Genes involved in the anthocyanin biosynthesis are: *chalcone synthase* (*CHS2*) **(A,B)**, and *UDP-glucose: flavonoid-3-O-glucosyltransferase* (*UFGT*) **(C,D)**. Gene expression was calculated during deficit treatment (I. During DI) and after re-irrigating vines with the original schedule (II. After DI). The error bars represent the standard deviation. Different letters on bars indicate significant differences among treatments and locations at *p* < 0.05 according to the Tukey HSD test within the same season. Different lower-case letters indicate a significant difference among treatments and locations in 2016 while capital letters are used for 2017.

*CHS* is a gene involved in the first step of anthocyanin biosynthesis. In both years, data showed an increase in *CHS2* gene expression in response to deficit irrigation treatments in both experimental sites (Figure 5A). Further, in Coachella Valley *CHS2* expression was higher compared to San Joaquin Valley after ending the DI treatment and re-irrigate vines with original schedule (Figure 5B). Further, *UFGT* is a major gene that controls anthocyanin biosynthesis in grapes (Kobayashi et al., 2001). Expression analysis revealed the induction of the *UFGT* as affected by the deficit irrigation treatments at both sites (Figure 5C). Interestingly, *UFGT* level during and after the DI treatments was higher in the San Joaquin Valley compared to the Coachella Valley (Figures 5C,D). Data also showed that in both sites, after ending the DI treatments and re-irrigating vines with the original schedule, *UFGT* expression increased as DI severity increased (Figure 5D). In addition, *flavanone 3-hydroxylase* (*F3H*) is another gene involved in the anthocyanin pathway; however, its transcripts were not induced significantly by DI treatments in both locations (Supplementary Figures 3A,B).

**FIGURE 6:**
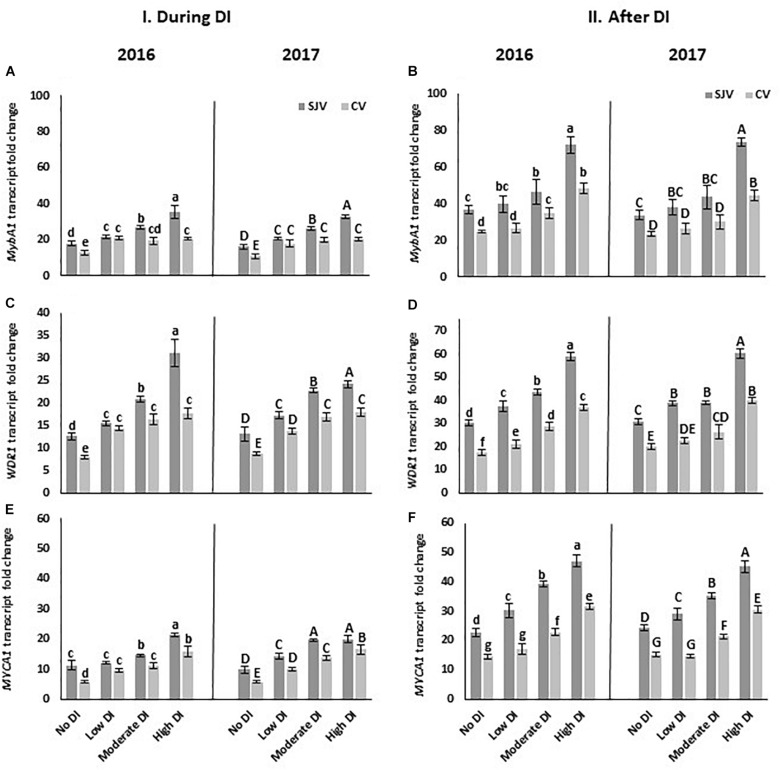
Expression of transcription factors involved in anthocyanin biosynthesis pathway in Scarlet Royal berry skin as affected by deficit irrigation (DI) treatments in 2016 and 2017, in San Joaquin (SJV) and Coachella (CV) Valleys. Transcription factors are: *MYBA1* **(A,B)**, *WDR1* **(C,D)**, and *MYCA1* **(E,F)**. Gene expression was calculated during deficit treatment (I. During DI) and after re-irrigating vines with the original schedule (II. After DI). The error bars represent the standard deviation. Different letters on bars indicate significant differences among treatments and locations at *p* < 0.05 according to the Tukey HSD test within the same season. Different lower-case letters indicate a significant difference among treatments and locations in 2016 while capital letters are used for 2017.

Moreover, *MYBA1*, is one of the transcription factors that respond to the external factors and activate *UFGT*. Data showed that the transcripts of *MYBA1* increased significantly in both years after applying the high DI treatment in both sites (Figure 6A). Interestingly, in San Joaquin Valley during DI treatments the expression of the *MybA1* was positively correlated with the severity of the DI treatments (Figure 6A). Similar to the *UFGT*, *MybA1* transcripts level was lower in Coachella Valley compared to San Joaquin Valley after ending the DI treatments and re-irrigating vines with the original schedule (Figure 6B). Further, DI activated the expression of the *tryptophan-aspartic acid repeat (WDR or WD40) protein* (*WDR1*) (Figures 6C,D) and the *basic helix-loop-helix (bHLH)* (*MYCA1*) (Figures 6E,F). Data also showed that the expression level of *WDR1* as well as *MYCA1*was lower in Coachella Valley compared to San Joaquin Valley after re-irrigating vines with the original schedule (Figures 6D,F).

### Gene Expression Analysis of Hormonal Biosynthesis Pathway

**FIGURE 7:**
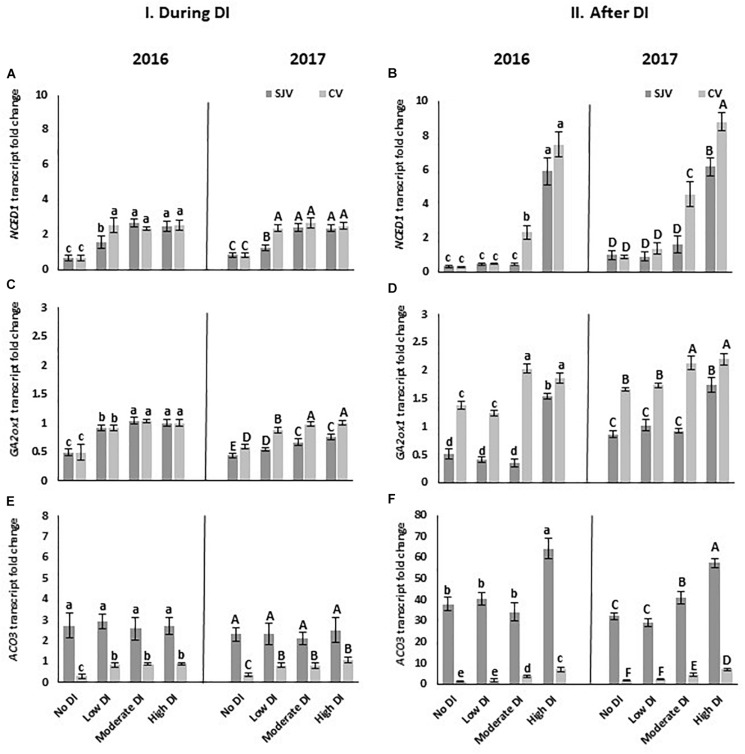
Expression of genes involved in hormonal biosynthesis pathway in Scarlet Royal berry skin as affected by deficit irrigation (DI) treatments in 2016 and 2017 in San Joaquin (SJV) and Coachella (CV) Valleys. Genes involved in the hormonal biosynthesis are: abscisic acid (ABA) biosynthesis gene, *9-cis-epoxycarotenoid dioxygenase* (*NCED1*) **(A,B)**, gibberellic acid (GA) catabolic gene *GA2 oxidase* (*GA2ox1*) **(C,D)**, and *1-aminocyclopropane-1-carboxylic acid oxidase* (*ACO3*) **(E,F)**. Gene expression was calculated during deficit treatment (I. During DI) and after re-irrigating vines with the original schedule (II. After DI). The error bars represent the standard deviation. Different letters on bars indicate significant differences among treatments and locations at *p* < 0.05 according to the Tukey HSD test within the same season. Different lower-case letters indicate a significant difference among treatments and locations in 2016 while capital letters are used for 2017.

The expression of some genes involved in different hormonal biosynthesis pathways was quantified using quantitative RT-qPCR (Figure 7). Data revealed the association of *NCED1*, and *ACO3* in the induction of anthocyanin biosynthesis. During DI, *NCED1* expression increased in response to DI treatments at both sites (Figure 7A). In San Joaquin Valley after re-irrigating the vines with the original irrigation schedule (after DI), the expression of *NCED1* declined in all treatments except for high DI (Figure 7B). However, after DI in Coachella Valley, *NCED1* expression remained higher in the moderate and high DI treatments compared to the other treatments (Figure 7B). Further, in 2017 the expression of this gene after ending the moderate and the high DI treatments was significantly higher in Coachella Valley compared to San Joaquin Valley (Figure 7B).

Data also showed that the transcript level of *GA2ox1*, gene catalyzes the 2-beta-hydroxylation of several biologically active gibberellins and controls the gibberellin inactivation pathway, was induced by the DI treatments (Figures 7C,D). The expression of this gene increased during DI in all treatments in both sites, however, its induction was higher in Coachella Valley compared to San Joaquin Valley in 2017 (Figure 7C). Moreover, after DI treatments, the increase of *GA2ox1* transcript was higher in Coachella Valley compared to San Joaquin Valley in all DI treatments in both years (Figure 7D).

The transcript level of *ACO3*, a major gene in ethylene biosynthesis, was also quantified. No induction of *ACO3* was observed during DI treatments in San Joaquin Valley. However, the expression of *ACO3* increased during DI in all treatments in Coachella Valley (Figure 7E). In both sites, a significant increase in *ACO3* expression in response to DI was observed at the moderate and high DI treatments after re-irrigating the vines with the original schedule (after DI) (Figure 7F). In general, the *ACO3* expression was significantly higher in San Joaquin Valley compared to Coachella Valley in all treatments including the well irrigated treatment (no DI).

### Gene Expression Analysis and Enzymes Activity of the Antioxidant System

**FIGURE 8:**
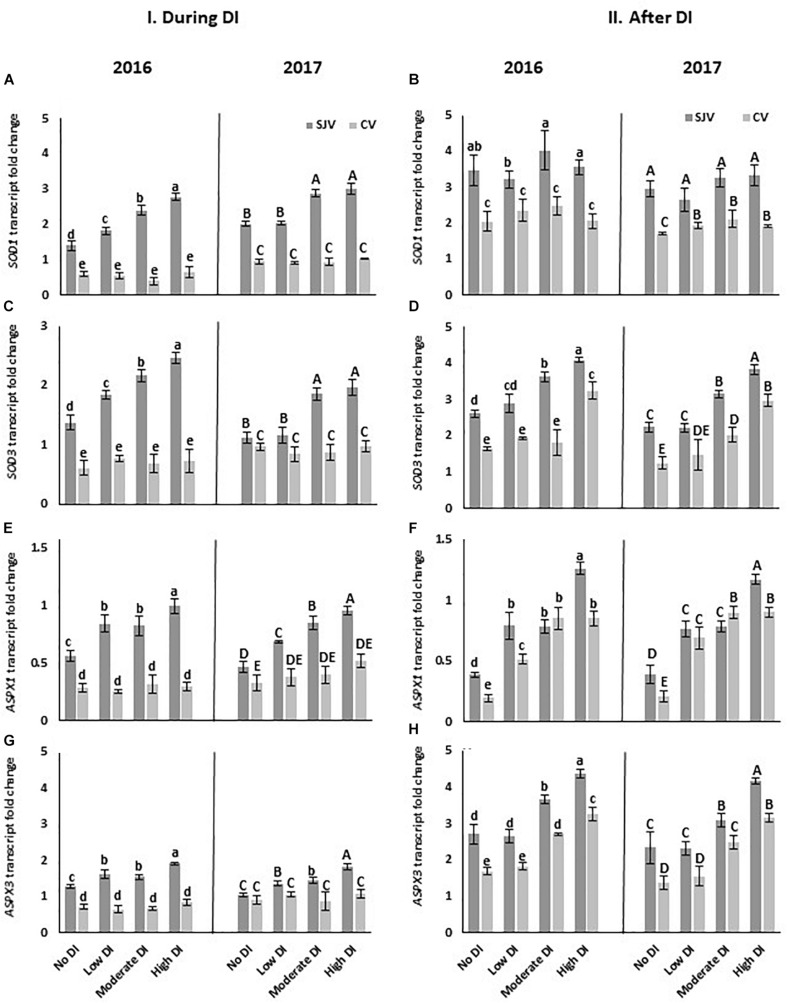
Expression of genes involved in the antioxidant system pathway in Scarlet Royal berry skin as affected by deficit irrigation (DI) treatments in 2016 and 2017 in San Joaquin (SJV) and Coachella (CV) Valleys. Genes involved in the antioxidant system are: *superoxide dismutase* (*SOD1* and *SOD3*) **(A–D)**, and *peroxidase* gene (*ASPX1* and *ASPX3*) **(E–H)**. Gene expression was calculated during deficit treatment (I. During DI) and after re-irrigating vines with the original schedule (II. After DI). The error bars represent the standard deviation. Different letters on bars indicate significant differences among treatments and locations at *p* < 0.05 according to the Tukey HSD test within the same season. Different lower-case letters indicate a significant difference among treatments and locations in 2016 while capital letters are used for 2017.

The expression of some genes involved in the antioxidant system such as superoxide dismutase (*SOD1* and *SOD3*) and peroxidase gene (*ASPX1* and *ASPX3*) were quantified using RT-qPCR. In general, it was noticed that the expression level of these genes in San Joaquin Valley was higher than in Coachella Valley (Figure 8). In the San Joaquin Valley, data showed that during DI, the moderate and high DI treatments significantly increased the expression of these four genes compared to the well-watered treatment (no DI) in both years (Figures 8A,C,E,G). However, in Coachella Valley during DI, the transcript level of these genes did not differ significantly between the well-watered treatment (no DI) and the DI treatments (Figures 8A,C,E,G). Data also revealed an increase in the expression of *SOD3*and *ASPX3* genes in response to the moderate and high deficit irrigation treatments at both experimental sites after ending the DI compared to the well-watered treatment (Figures 8D,H).

**FIGURE 9:**
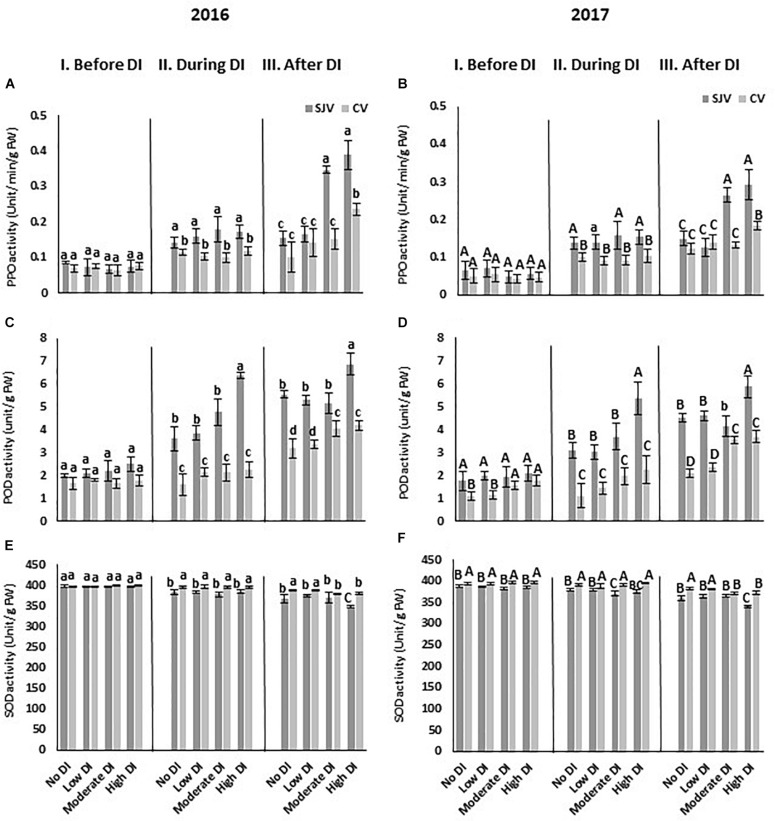
Antioxidant enzymes activity in Scarlet Royal berry skin as affected by deficit irrigation (DI) treatments in 2016 and 2017 in San Joaquin (SJV) and Coachella (CV) Valleys. Enzymes involved in the antioxidant system are: polyphenol oxidase (PPO) **(A,B)**, peroxidase (POD) **(C,D)**, and superoxide dismutase (SOD) **(E,F)**. Enzyme activity was measured before deficit (I. Before DI), during deficit (II. During DI) and after re-irrigating vines with the original schedule (III. After DI). The error bars represent the standard deviation. Different letters on bars indicate significant differences among treatments and locations at *p* < 0.05 according to the Tukey HSD test within the same season. Different lower-case letters indicate a significant difference among treatments and locations in 2016 while capital letters are used for 2017.

The activity of the enzymes involved in the antioxidant system such as PPO, POD, and SOD was also quantified (Figure 9). Data showed that, the enzyme activity of PPO was similar in both locations and it did not change significantly during DI among treatments in both sites (Figures 9A,B). In San Joaquin Valley, after re-irrigating the vines with the original schedule (after DI), PPO activity increased significantly in the moderate and high DI treatments (Figures 9A,B). However, in Coachella Valley the increase in PPO activity only occurred after ending the high DI treatments in both years (Figures 9A,B). Moreover, the enzyme activity of POD showed a lower level of activity in the Coachella Valley before DI in the no DI and low DI treatments compared to the San Joaquin Valley only in 2017 (Figure 9D). During DI treatments, POD increased following the high DI in San Joaquin Valley only (Figures 9C,D). After ending the DI treatment, the POD increased to its highest level following the high DI treatment in San Joaquin Valley (Figures 9C,D). However, in Coachella Valley, after re-irrigating the vines with the original schedule (after DI), POD activity increased significantly in the moderate and high DI treatments (Figures 9C,D). In both sites, SOD activity did not change significantly during DI among treatments (Figures 9E,F). However, during DI, SOD activity was higher significantly in Coachella Valley compared to San Joaquin Valley among all treatments (Figures 9E,F). After DI treatments, SOD level decreased significantly by the high DI treatment in the San Joaquin Valley, and by the moderate and high DI treatments in the Coachella Valley compared to the other treatments (Figures 9E,F).

### *MYBA1* and *UFGT* Promoter Sequences Analysis

*MYBA1* is one of the transcription factors that respond to the external factors and activate *UFGT* to stimulate anthocyanin biosynthesis during grape ripening (Azuma et al., 2007, 2008). Induction of the expression of these genes is controlled by the regulatory elements present in the promoter region. The sequence of the *MYBA1* promoter was obtained from http://www.ncbi.nlm.nih.gov and used for promoter analysis using the PlantCARE website^1^ (Lescot et al., 2002) and PLACE website^2^ (Higo et al., 1999). Gene sequences analysis of the promoter region of *MYBA1* revealed that this genes is controlled by several internal and environmental factors (Supplementary Figure 4). Further, Chervin et al. (2009) previously reported similar finding in the *UFGT* promoter. Promoters of these two genes possess several *cis*-elements for light, ethylene, abscisic acid, methyl jasmonate, sugars, and salicylic acid. Some of these compounds are in use already in the vineyards, such as ABA and ethylene; however, the others need to be tested.

## Discussion

Data has shown that anthocyanin accumulation is lower under the Coachella Valley conditions compared to the San Joaquin Valley. This data was confirmed by visually evaluating the berry color, measuring the total anthocyanin spectrophotometry in berry skin, and identifying the individual anthocyanin compounds by using HPLC analysis. Interestingly, we observed that Mv-3-glc, Mv-3-cmg, and Mv-3-cmglc were detected in very low quantities in the Coachella Valley compared to the San Joaquin Valley. In fact, except for Cn-3-glc, all the quantified anthocyanins are in general lower in samples from the Coachella Valley, regardless of treatment. This indicates a widespread negative impact on the anthocyanin pathways that are likely due to the growing conditions at this location. Individual anthocyanin compound followed different trends following DI treatments, however, it is clear that the response is different from year to year and that could be due to the changes in the weather and the cultural practices applied in the vineyard. It seems that some compounds such as Pn-3-glc that are induced by the low DI level in San Joaquin Valley required high DI treatment to be induced in Coachella Valley. Other compounds are not induced by the DI in Coachella Valley, such as Cn-3-glc and Mv-3-cmg. It seems that, the lack of anthocyanin accumulation in Coachella Valley could be due to the negative effect of the high temperature on the synthesis of these compounds as suggested by Mori et al. (2007). The increase in the anthocyanin content is unlikely to be due to the reduction in berry size since none of the treatments caused a significant reduction in berry size (Supplementary Table 1).

The induction of anthocyanin accumulation following DI treatments in both sites was associated with higher expression of key genes involved in anthocyanin synthesis such as *UFGT*, *MYBA1*, *MYCA1*, and *WDR1*. However, their expression was lower in Coachella Valley compared to San Joaquin Valley. The transcription factors *MYBA1* and *MYCA1* form a complex together to activate the anthocyanin biosynthesis pathway in grapes (Jiu et al., 2021), lower transcripts level of these genes in Coachella could be one of the major reasons for the lower anthocyanin level at this location. The low expression of *MYBA1*, *MYCA1*, and *WDR1* in Coachella Valley indicates that other factors, such as high temperature, may affect the expression of these genes and consequently affect the *UFGT* gene expression. These transcription factors play a major role in regulating the anthocyanin biosynthesis in grapes (Azuma et al., 2011; Jaakola, 2013; Jiu et al., 2021). Inactivation of the expression of the *MYBA1* affects the anthocyanin biosynthesis (Kobayashi et al., 2005). The obtained data demonstrated that deficit irrigation regulates the anthocyanin pathway at least partially at the transcriptional level. Accumulation of the sugars in the berry during the maturation process is involved in the biosynthesis and accumulation of anthocyanins in grape berries (Boss and Davies, 2009). Our data showed that berry sugar content increased in all DI treatments in both locations, however, it became significant in the moderate and high DI treatments and was significantly higher in San Joaquin Valley location (Supplementary Table 1).

Our data also indicated an increase in *CHS2* in response to DI treatments at both sites. However, after DI and irrigating vines with the original schedule, its expression was higher in Coachella Valley compared to San Joaquin Valley. Besides its role in anthocyanin biosynthesis, *CHS* is also known to be involved in responding to different stress conditions such as heat stress (Wu et al., 2020). Thus, the high expression of this gene in Coachella Valley after DI could be due to its induction by other environmental stress conditions. ABA is a stress hormone which was shown to be induced following DI treatments (Rossdeutsch et al., 2016). It is also involved in inducing the expression of genes involved in the anthocyanin biosynthesis pathway (Ban et al., 2003). We used the *NCED1* gene expression as an indicator for the activation of ABA biosynthesis and the transcripts level of that gene correlated well with the severity of water stress applied by DI in both locations. The *NCED1* transcript profile looks similar in both locations during the DI treatment. This data indicates that the level of ABA required to activate the anthocyanin pathway in both locations was similar. Further, *ACO3* oxidase, which is involved in ethylene biosynthesis, increased as the severity of DI increased in both locations. Its level, however, was lower in the Coachella Valley, suggesting the need for external application to boost the anthocyanin pathway. Some hormonal treatment may be needed to fully activate the anthocyanin pathway in the Coachella Valley.

Our data collectively indicate that vines located in Coachella Valley are under stress compared to those in San Joaquin Valley, as is shown by the higher expression of the *GAox2* gene. This gene was reported to be involved in stress tolerance since the mutant of this gene enhances *Arabidopsis* abiotic stress tolerance (Lo et al., 2017). Another indicator for the stress in Coachella Valley is the higher *CHS2* gene transcript level, lower transcript level of *SOD* and *ASPX* antioxidant genes, and lower PPO and POD enzyme activity. *SOD* and *ASPX* genes remove free radicals (reactive oxygen species) produced under various environmental stresses. The lack of expression of these two genes suggests a lower antioxidant capacity in vines grown at Coachella Valley. Consequently, an imbalance of the generation and detoxification of ROS might occur (Hasanuzzaman et al., 2020). Therefore, the accumulation of free radicals results in impairing anthocyanin biosynthesis (Movahed et al., 2016). This might have a negative role on the anthocyanin biosynthesis pathway.

The function of antioxidant enzymes such as PPO, POD, and SOD are critical in alleviating abiotic stress. The lower level of these enzymes in the Coachella Valley may have failed to protect the anthocyanin biosynthesis and reduced the efficiency of DI to induce anthocyanin accumulation. This data suggested that the lack of red coloration could be at least partially due to the lower level of antioxidant activities which allow for the accumulation of free radicals. These free radicals can result in accelerated anthocyanin degradation and impaired anthocyanin biosynthesis (Movahed et al., 2016). Potential treatments could be designed to boost the antioxidant enzyme activity in order to improve anthocyanin accumulation under this condition in combination with DI treatments. These treatments could include anti-stress products to help vines overcome the damaging effect of free radicals. This hypothesis is valid, we recently reported that zinc-nanoparticles increased the SOD enzyme activity, anthocyanin accumulation and improved berry color in Crimson Seedless table grape (Abou El-Nasr et al., 2021). Taking in consideration of the results of the present study, similar more inclusive treatments that include more than one factor could be tested in the vineyard to improve anthocyanin accumulation in table grapes.

The Coachella Valley is characterized by desert-like weather conditions, including high spring and summer temperatures (Table 1). It is evident that higher temperature close to 35°C inhibits plant metabolism, including anthocyanin biosynthesis (Mori et al., 2007). High temperature impairs the antioxidant activity system which negatively affects the anthocyanin biosynthesis, consequently leading to reduced red coloration in Coachella Valley. In addition, gene sequences analysis for the promoter region of *UFGT* and *MYBA1*, two key genes in anthocyanin biosynthesis, was previously reported to have cis element for light and hormones, revealing that these genes are controlled by several internal and environmental factors such as light. It has been reported that light is an important factor for plant pigmentation, including anthocyanin (Blancquaert et al., 2019). Some cultural practices already exist in table grape vineyards to improve light penetration through the canopy and improve grape coloration, including shoot and leaf removal (Jeong et al., 2004; Tarricone et al., 2020).

Generally, it seems that ABA and ethylene are essential to accumulate anthocyanins, suggesting an important role for the exogenous application of ethephon and ABA at color break stage (veraison). It is well known that endogenous ethylene and ABA evolution around veraison plays an important role in establishing grape berry ripening (Coombe and Hale, 1973; Coombe, 1976). In addition, exogenous application of the ethylene and ABA enhances anthocyanin accumulation and improves berry coloration (El-Kereamy et al., 2003; Balint and Reynolds, 2013; Leão et al., 2015). Our data suggested that these treatments could be more effective if combined with other cultural practices that increase light surrounding the clusters or/and improve antioxidant activities. Further, the induction of the *GA2 oxidase*, which is involved in GA degradation, indicates a possible role for GA in this process (Sakamoto et al., 2001). GA and/or GA inhibitor in combination with DI treatment could be tested to check their effect on anthocyanin accumulation in grapes.

## Conclusion

The current study provides valuable information to be used to improve table grape coloration, especially under challenging high temperature. It seems that warmer weather conditions affect the vines’ response to deficit irrigation and consequently negatively affects the DI outcome in improving anthocyanin accumulation. Under unfavorable weather condition other factors need to be considered for effective deficit irrigation treatment. Our data showed that anthocyanin accumulation following the DI treatment was lower under warmer conditions and it is associated with a lower level of anthocyanin biosynthesis and antioxidant gene expression. These genes are controlled by several endogenous and environmental factors. It is suggested that these enzymes may be important in alleviating the stress and improving anthocyanin accumulation in table grapes. Based on the obtained results, two approaches could be proposed to improve the accumulation of the red pigment in table grapes. The first approach is to reduce the negative effect of the environmental stress through stimulation of the antioxidant pathway to help remove the free radicals. The second approach involves improving the expression of critical genes involved in the anthocyanin pathway through hormonal treatments or modifying the light spectrum around the clusters. Our data indicated that several factors are associated with the lower accumulation of anthocyanin in table grapes. To improve the red color of table grapes, deficit irrigation could be used, however, other factors need to be optimized, such as light perception, hormonal balance, and antioxidant activities. Deficit irrigation is not the only factor that regulates anthocyanin accumulation in table grapes.

## Data Availability Statement

The original contributions presented in the study are included in the article/Supplementary Material, further inquiries can be directed to the corresponding author/s.

## Author Contributions

MA performed field work, sample collections, data analysis, and prepared the manuscript. DO performed HPLC analysis and revised the manuscript. AE-K performed laboratory analysis and edited the manuscript. All authors contributed to the article and approved the submitted version.

## Conflict of Interest

The authors declare that the research was conducted in the absence of any commercial or financial relationships that could be construed as a potential conflict of interest.

## Publisher’s Note

All claims expressed in this article are solely those of the authors and do not necessarily represent those of their affiliated organizations, or those of the publisher, the editors and the reviewers. Any product that may be evaluated in this article, or claim that may be made by its manufacturer, is not guaranteed or endorsed by the publisher.

## Footnotes

1
http://bioinformatics.psb.ugent.be/webtools/plantcare/html/

2
https://www.dna.affrc.go.jp/PLACE/?action=newplace

## Supplementary Material

Supplementary Figure 1 Irrigation treatments imposed for Scarlet Royal vines grown in San Joaquin **(A)** and Coachella **(B)** Valleys in 2016 and 2017.

Supplementary Figure 2 Leaf water potential values of Scarlet Royal vines grown in San Joaquin (SJV) and Coachella (CV) Valleys as affected by different deficit irrigation (DI) treatments. Measurements were taken in 2016 **(A)** and 2017 **(B)** before deficit (Before DI), during deficit (During DI) and after re-irrigating vines with the original schedule (After DI). The error bars represent the standard deviation. Different letters on bars indicate significant differences among treatments and locations at *p* < 0.05 according to the Tukey HSD test within the same season. Different lower-case letters indicate a significant difference among treatments and locations in 2016 while capital letters are used for 2017.

Supplementary Figure 3 Expression of *Flavanone 3-hydroxylase* (*F3H*) in Scarlet Royal berry skin as affected by deficit irrigation (DI) treatments in 2016 and 2017, in San Joaquin (SJV) and Coachella (CV) Valleys. Gene expression was calculated during deficit treatment during DI **(A)** and after re-irrigating vines with the original schedule **(B)**. The error bars represent the standard deviation. Different letters on bars indicate significant differences among treatments and locations at *p* < 0.05 according to the Tukey HSD test within the same season. Different lower-case letters indicate a significant difference among treatments and locations in 2016 while capital letters are used for 2017.

Supplementary Figure 4 Different cis-elements detected in the *MYBA1* promoter region from −2323 to +1. Sequence was analyzed using the PlantCARE website (http://bioinformatics.psb.ugent.be/webtools/plantcare/html/; Lescot et al., 2002) and PLACE website (https://www.dna.affrc.go.jp/PLACE/?action=newplace; Higo et al., 1999). Number of lines indicates the nucleotide location relative to the start codon. Cis-elements sequence are indicated in bold letters and abbreviations of the different responsive elements are ABA (ABRE), ethylene (ERE), light (LRE), sugar (SURE), methyl jasmonate (JARE), gibberellic acid (GARE), auxin (AuxRE), and salicylic acid (SARE).

Supplementary Table 1 Scarlet Royal berry sugar, acidity, weight and size as affected by deficit irrigation (DI) treatments in San Joaquin (SJV) and Coachella (CV) VALLEYs. Samples were collected at harvest time in 2016 and 2017. Each value indicates the mean of four replicates ± its standard deviation. Different letters indicate significant differences among treatments and locations at *p* < 0.05 according to the Tukey HSD test within the same season. Different lower-case letters indicate a significant difference among treatments and locations in 2016 while capital letters are used for 2017.

Supplementary Table 2 Primers for genes used for RT-qPCR.
