# Supplementary material for: The Complexity of Modulating Anthocyanin Biosynthesis Pathway by Deficit Irrigation in Table Grapes
Source: Front Plant Sci. 2021 Aug 18;12:713277. doi: 10.3389/fpls.2021.713277 (PMC8416356; doi:10.3389/fpls.2021.713277)
Supplement: Supplementary Figure 1 — Irrigation treatments imposed for Scarlet Royal vines grown in San Joaquin (A) and Coachella (B) Valleys in 2016 and 2017. [file Data_Sheet_1.PDF]

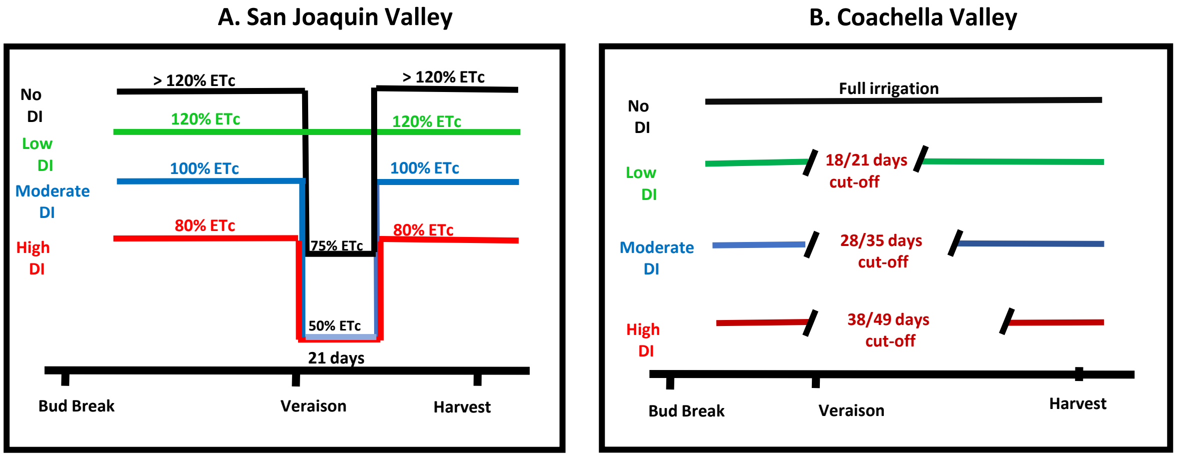

Supplementary Figure 1. Irrigation treatments imposed for Scarlet Royal vines grown in San Joaquin (A) and Coachella (B) valleys in 2016 and 2017.
