# Supplementary material for: The Complexity of Modulating Anthocyanin Biosynthesis Pathway by Deficit Irrigation in Table Grapes
Source: Front Plant Sci. 2021 Aug 18;12:713277. doi: 10.3389/fpls.2021.713277 (PMC8416356; doi:10.3389/fpls.2021.713277)
Supplement: Supplementary Figure 2 — Leaf water potential values of Scarlet Royal vines grown in San Joaquin (SJV) and Coachella (CV) Valleys as affected by different deficit irrigation (DI) treatments. Measurements were taken in 2016 (A) and 2017 (B) before deficit (Before DI), during deficit (During DI) and after re-irrigating vines with the original schedule (After DI). The error bars represent the standard deviation. Different letters on bars indicate significant differences among treatments and locations at p < 0.05 according to the Tukey HSD test within the same season. Different lower-case letters indicate a significant difference among treatments and locations in 2016 while capital letters are used for 2017. [file Data_Sheet_2.PDF]

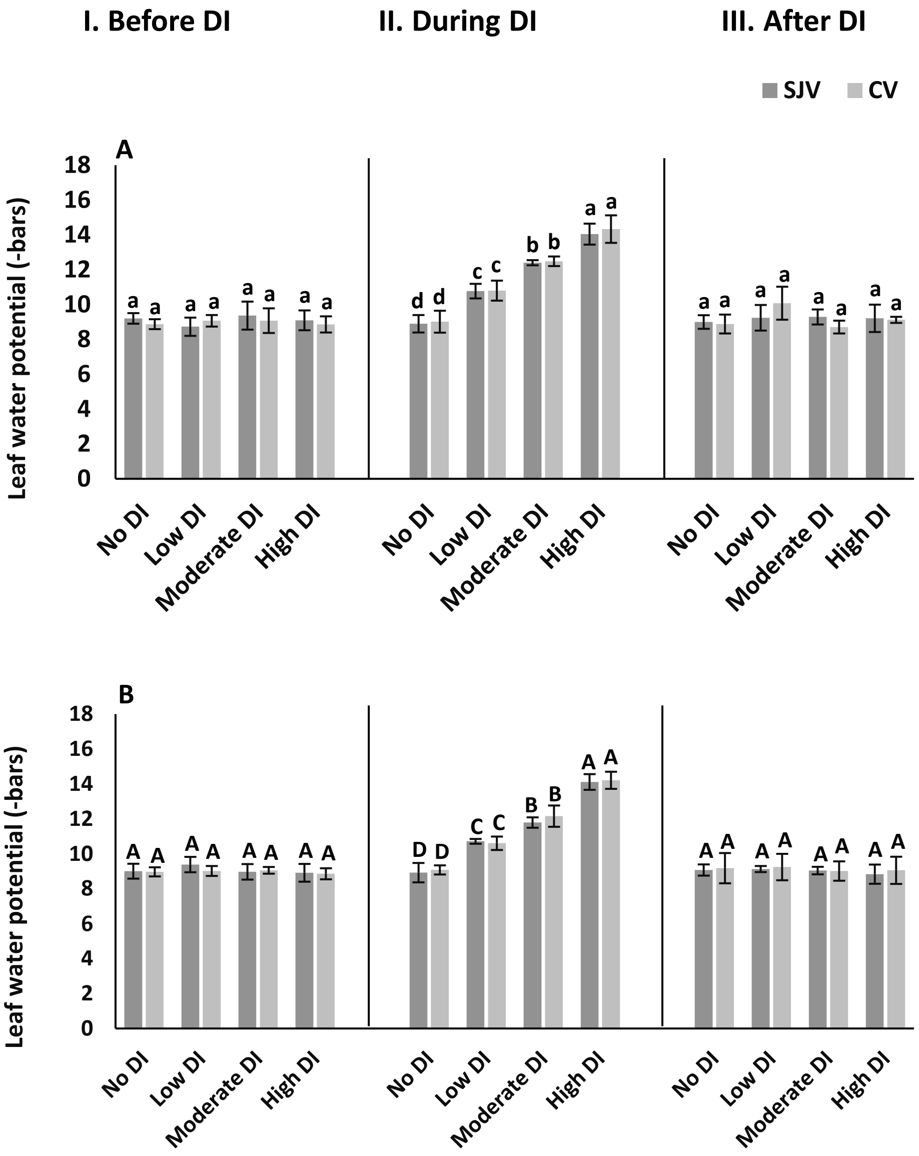

Supplementary Figure 2. Leaf water potential values of Scarlet Royal vines grown in San Joaquin (SJV) and Coachella (CV) valleys as affected by different deficit irrigation (DI) treatments. Measurements were taken in 2016 (A) and 2017 (B) before deficit (Before DI), during deficit (During DI) and after re-irrigating vines with the original schedule (After DI). The error bars represent the standard deviation. Different letters on bars indicate significant differences among treatments and locations at $p < 0.05$ according to the Tukey HSD test within the same season. Different lower-case letters indicate a significant difference among treatments and locations in 2016 while capital letters are used for 2017.
