# Supplementary material for: The Complexity of Modulating Anthocyanin Biosynthesis Pathway by Deficit Irrigation in Table Grapes
Source: Front Plant Sci. 2021 Aug 18;12:713277. doi: 10.3389/fpls.2021.713277 (PMC8416356; doi:10.3389/fpls.2021.713277)
Supplement: Supplementary Figure 3 — Expression of Flavanone 3-hydroxylase (F3H) in Scarlet Royal berry skin as affected by deficit irrigation (DI) treatments in 2016 and 2017, in San Joaquin (SJV) and Coachella (CV) Valleys. Gene expression was calculated during deficit treatment during DI (A) and after re-irrigating vines with the original schedule (B). The error bars represent the standard deviation. Different letters on bars indicate significant differences among treatments and locations at p < 0.05 according to the Tukey HSD test within the same season. Different lower-case letters indicate a significant difference among treatments and locations in 2016 while capital letters are used for 2017. [file Data_Sheet_3.PDF]

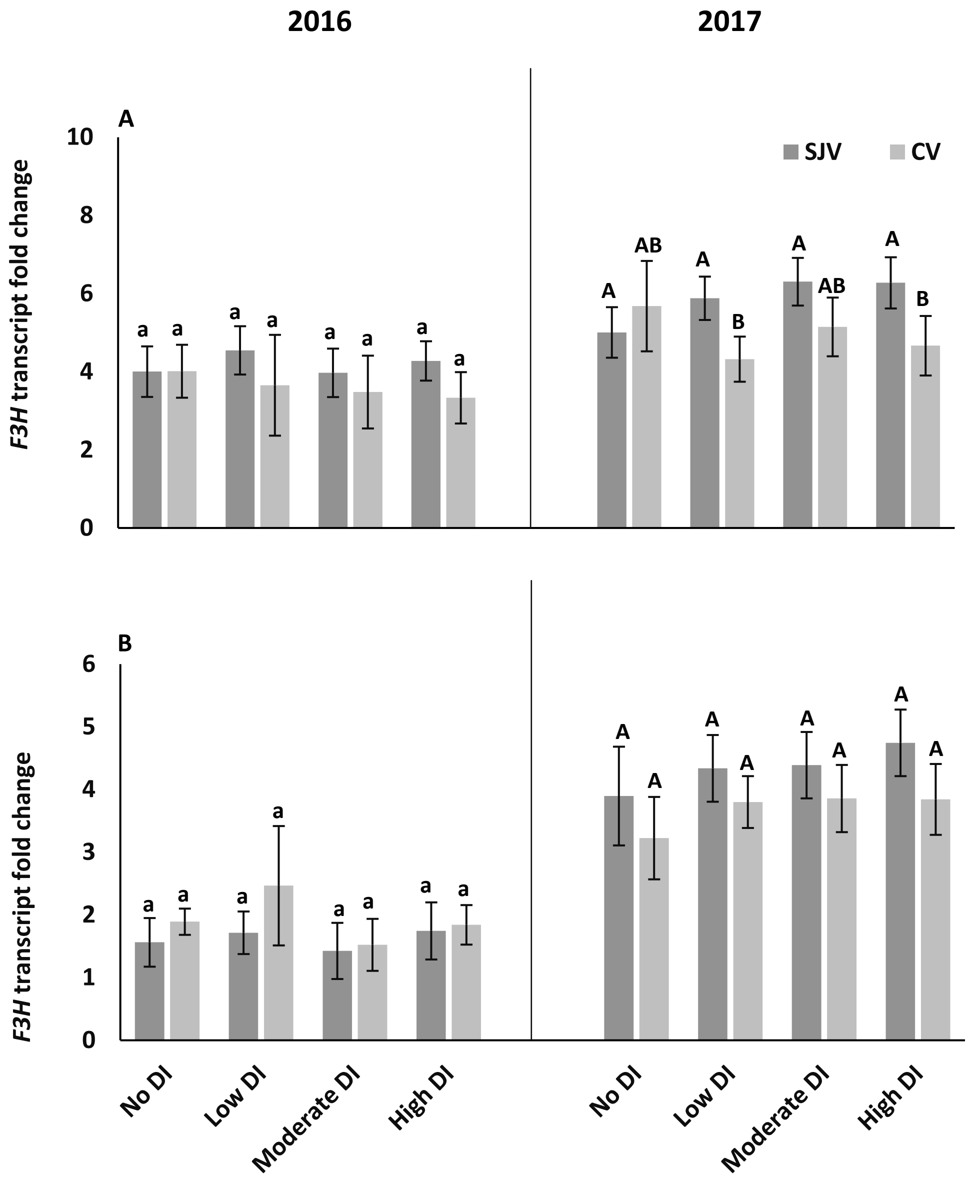

Supplementary Figure 3. Expression of Flavanone 3-hydroxylase (*F3H*) in Scarlet Royal berry skin as affected by deficit irrigation (DI) treatments in 2016 and 2017, in San Joaquin (SJV) and Coachella (CV) valleys. Gene expression was calculated during deficit treatment during DI (A) and after re-irrigating vines with the original schedule (B). The error bars represent the standard deviation. Different letters on bars indicate significant differences among treatments and locations at $p < 0.05$ according to the Tukey HSD test within the same season. Different lower-case letters indicate a significant difference among treatments and locations in 2016 while capital letters are used for 2017.
