# Supplementary material for: The Complexity of Modulating Anthocyanin Biosynthesis Pathway by Deficit Irrigation in Table Grapes
Source: Front Plant Sci. 2021 Aug 18;12:713277. doi: 10.3389/fpls.2021.713277 (PMC8416356; doi:10.3389/fpls.2021.713277)
Supplement: Supplementary Figure 4 — Different cis-elements detected in the MYBA1 promoter region from −2323 to +1. Sequence was analyzed using the PlantCARE website (http://bioinformatics.psb.ugent.be/webtools/plantcare/html/; Lescot et al., 2002) and PLACE website (https://www.dna.affrc.go.jp/PLACE/?action=newplace; Higo et al., 1999). Number of lines indicates the nucleotide location relative to the start codon. Cis-elements sequence are indicated in bold letters and abbreviations of the different responsive elements are ABA (ABRE), ethylene (ERE), light (LRE), sugar (SURE), methyl jasmonate (JARE), gibberellic acid (GARE), auxin (AuxRE), and salicylic acid (SARE). [file Data_Sheet_4.PDF]

ABRE

-2323 TGCTGAGACAGCCAAAGGACGCCTTCTCCACCCAGTCCACAAG**GTGGC**CCCTCAATTCCGGACCTACATT

AuxRE

-2253 AA**CAGCAA**CAAAAGATGAGAATGCCATCCGGAAGAGTCAACAAAAAATCAACAAAGTAAAAACCGGACAA

-2183 TAGTCACAAAGCAAACCTACCCGGAACCACCAAGGAGTAGTTTGGAGAGAAAGGCCTCGCCGGATGCTCC

LRE ABRE ABRE

-2113 AAAAGCC**CCGCCC**ATGCACCCCGG**ACCACCACGTGTCCCGTCG**CCCCTCCCTTTGTCGAATCTGGCAGCT

JARE JARE

-2043 C**CGTCACC**AGTTGGAGGGAGGGCAGGTGTGCGGACATGCTGAAGATGTCATTTTTGACCTTCTTCAAAGA

-1973 ATACACAAAAAACACCTCCAGCAGCGTGAGATCGAGGTTGTACAGCATGTTTATGATGCTGCATCCCATC

GARE SURE

-1903 AGCACCCGGACGATGT**TGGGAT**G**AATAAA**GACGGGTGGAATCTGGGTGAAGTGGAGGAACTCCTTGAACA

-1833 ACGCCAGCAGAGGGAACCGGAGCCCCGCGTTGAATTGTTCCTTCGAGAAGATGATAGTTTTTTCTTCTGC

AuxRE JARE ABRE LRE

-1763 TTTCTCAGAAGGAACAAGCACCCTCCCATT**CAGCAA**TTCGATAGCCA**CGTCATTTGG**GATACAGAACCGT

LRE

-1693 TCCCGGAATTCCTTCGCGTCCAAC**TTATCTAT**CGCTTTTCCACGAACCTCGGTACCCCGAGAAGATGAAA

LRE

-1623 CAGTCTTTTTTGCAGCCATT**TCTTAC**CACAAACACAAGCCCAAAACTACACACAAGAAGCGCAATGGGTA

-1553 AAACACAAGTAACCCAGAAAATACACAAACCCTAACCCGAACAAATCAGAAACCAAAACCAACAAAACGC

GARE AuxRE ABRE LRE ABRE

-1483 AA**CAAAAGG**AA**CAGCAA**TAGCAAAAGTA**CACGTACC**AAATGAAGCTCTGAAGAAGAAAGGTCGTCGTTTC

AuxRE

-1413 GAATACAAGAACACCAG**CAGCAA**ACAAATGTCGCGACACAAAATCACCGGTACAAAAGTGTTTTCGAGTT

-1343 TCTTTACTCAGAAAAAGCAAAGGACGCAACAAAAGCAAGAAGAAGAAAGGATCTCAGGAAAATGCAGTAG

SARE

-1273 AAAAAATG**AAAAAGAAGG**TACAGTACGATATTTATAGGGGGACCAGCCCTCGGAAAGCCAAACGTCCAAA

-1203 CAGCGCTATCATTGAAACAACATGCTGCCTGGGAATTCCCAGAGTCGCCGGCTCATCATAAATGCCTTTT

ABRE LRE ABRE LRE

-1133 ATGGCTTCCGCACCCCCTCTCG**CCACGTGGCTCAG**TCAGACGAAGAGACCTCTTCAAAATTCAAAAAGCC

SURE LRE

-1063 AG**TTATTT**T**TTAACC**CGCCCATTTTTTGGCAAAATAGGCAAGTTAAAAAGGGGGGCAATGTAGGGACCCC

LRE ABRE LRE — LRE ABRE

-0993 TCCCTCTGGGAAA**CACGTGGCAC**GCACCTCACAGTGACACGCAG**CACGTGTTAT**CAGCCGGACCATCATC

LRE

-0923 ATCCGGATTCCCTTAAGGATACGCATGATGATGGTTC**TCCTATC**CGGACCGCCTCAAGGAAAAGCACA**TG**

JARE AuxRE

-0853 **ACG**TTTCAGCTTCTTCTGTCCAAGGAAGAGCAAACGACGCTGACAGAGCATAGACATCCGGACAACCTTC

-0783 ATAATGTATCTGCTCCACTATACAATCCGGATAGTCAGCATGTGACCATCCGGATTTAATCGTCCGGATC

LRE LRE

-0713 ATCAATTAAAGTAAAGCAAG**TCTTAC**ACGCTAT**CACGAC**AACCAGCCATGGCCCACATCCCATCATCTGC

-0643 AGAGTGAAAGGACGGGTCGAGGTGACAACAAGTCACTTCCCACGATCATTCTACATGATCATTTCCCACG

ERE ABRE

-0573 ATATCTAGACAGCAGCATCACCTACCACGGTTTCTGACA**GCCGCC**AGTAGG**GTGGC**GATGACCATGCTGC

LRE

-0503 CTCCGAATGTCATCATGACAAACATAAAATATCTCCTCGCC**ATTAAT**GAGAGGAACAGTACCCCTGAAGC

-0433 TGTATATATATGCCTTCGCACGAAGAAGAAGGGGATCCTCCTGGTAACTTCTTAATACCTGGTAAAAGGC

-0363 CAACTAATTTATATTCCTCTCTCTAACCATGGCTAACAAAACCATCGGAGGATGCGTCCAGACACCCTGT

JARE

-0293 CCGGATGCCTTCTTGCAGGAA**TGACG**ACTGGATCAAAAACCTTTATGAGTTGAGATCACGCGTCCATCCA

ABRE/LRE

-0223 TCTGGTTAC**TACGTG**GACCGCCAAAGACGCGAGGTAACAACAACACACACCCTTTGTCCATGAACTCCAG

LRE ABRE LRE SURE

-0153 CGC**ATTTGG**AAGCCAGTAATGCACCATAA**GAAACGTGTC**G**AATAAA**CCAATTAGGGGTCTGGTGTCCGAG

TATA Box

-0083 TCATGAGATAGAACAGGTTCGAGGTTGT**TATA**TATCAATCAATAATTAGAGAAGGAGCCGGTCTCTTGTG

-0013 TTGAGTTGACTCG

Supplementary Figure 4. Different cis-elements detected in the *MYBA1* promoter region from -2323 to +1. Sequence was analyzed using the PlantCARE website (http://bioinformatics.psb.ugent.be/webtools/plantcare/html/) (Lescot et al., 2002) and PLACE website (https://www.dna.affrc.go.jp/PLACE/?action=newplace) (Higo et al., 1998). Number of lines indicates the nucleotide location relative to the start codon. Cis-elements sequence are indicated in bold letters and abbreviations of the different responsive elements are ABA (ABRE), ethylene (ERE), light (LRE), sugar (SURE), methyl jasmonate (JARE), gibberellic acid (GARE), auxin (AuxRE) and salicylic acid (SARE).
