# Supplementary material for: The Complexity of Modulating Anthocyanin Biosynthesis Pathway by Deficit Irrigation in Table Grapes
Source: Front Plant Sci. 2021 Aug 18;12:713277. doi: 10.3389/fpls.2021.713277 (PMC8416356; doi:10.3389/fpls.2021.713277)
Supplement: Supplementary Table 1 — Scarlet Royal berry sugar, acidity, weight and size as affected by deficit irrigation (DI) treatments in San Joaquin (SJV) and Coachella (CV) VALLEYs. Samples were collected at harvest time in 2016 and 2017. Each value indicates the mean of four replicates ± its standard deviation. Different letters indicate significant differences among treatments and locations at p < 0.05 according to the Tukey HSD test within the same season. Different lower-case letters indicate a significant difference among treatments and locations in 2016 while capital letters are used for 2017. [file Data_Sheet_5.pdf]

Supplementary Table 1. Scarlet Royal berry sugar, acidity, weight and size as affected by deficit irrigation (DI) treatments in San Joaquin (SJV) and Coachella (CV) valleys. Samples were collected at harvest time in 2016 and 2017. Each value indicates the mean of four replicates ± its standard deviation. Different letters indicate significant differences among treatments and locations at $p < 0.05$ according to the Tukey HSD test within the same season. Different lower-case letters indicate a significant difference among treatments and locations in 2016 while capital letters are used for 2017.

	2016							
	No DI		Low DI		Moderate DI		High DI	
	SJV	CV	SJV	CV	SJV	CV	SJV	CV
**Berry sugar content (°Brix)**	18.7 ± 0.57 **b**	17 ± 0.29 **c**	19.2 ± 0.29 **b**	18 ± 1.00 **bc**	20.5 ± 0.37 **a**	19.1 ± 0.89 **b**	21.2 ± 0.61 **a**	19.5 ± 0.25 **b**
**Titratable acidity (g/100 mL)**	0.52 ± 0.01 **a**	0.63 ± 0.02 **c**	0.45 ± 0.02 **b**	0.56 ± 0.01 **d**	0.45 ± 0.01 **bc**	0.54 ± 0.03 **d**	0.44 ± 0.03 **bc**	0.56 ± 0.02 **d**
**Juice pH**	3.80 ± 0.03 **a**	3.84 ± 0.06 **a**	3.81 ± 0.06 **a**	3.84 ± 0.04 **a**	3.80 ± 0.07 **a**	3.80 ± 0.07 **a**	3.86 ± 0.01 **a**	3.85 ± 0.06 **a**
**Berry weight (g)**	7.75 ± 1.12 **a**	6.80 ± 0.39 **a**	7.55 ± 0.42 **a**	6.66 ± 0.70 **a**	7.96 ± 0.23 **a**	6.91 ± 0.76 **a**	7.89 ± 0.40 **a**	7.02 ± 0.52 **a**
**Berry length (mm)**	28.72 ± 0.60 **a**	27.75 ± 0.33 **a**	28.10 ± 1.25 **a**	27.40 ± 0.96 **a**	28.19 ± 0.69 **a**	26.85 ± 1.10 **a**	28.15 ± 0.60 **a**	27.95 ± 0.47**a**
**Berry width (mm)**	21.08 ± 0.62 **a**	20.10 ± 0.51 **a**	20.65 ± 0.57 **a**	19.87 ± 0.71 **a**	21.11 ± 0.23 **a**	19.32 ± 0.54 **a**	21.17 ± 0.38 **a**	20.23 ± 0.49 **a**
	2017							
	No DI		Low DI		Moderate DI		High DI	
	SJV	CV	SJV	CV	SJV	CV	SJV	CV
**Berry sugar content (°Brix)**	18.6 ± 0.61 **B**	15.9 ± 0.55 **C**	19.3 ± 0.15 **B**	17 ± 0.91 **BC**	20.8 ± 0.64 **A**	17.5 ± 0.55 **BC**	21.1 ± 0.43 **A**	18.6 ± 0.91 **B**
**Titratable acidity (g/100 mL)**	0.68 ± 0.06 **A**	0.59 ± 0.01 **A**	0.58 ± 0.03 **B**	0.5 ± 0.05 **C**	0.61 ± 0.02 **AB**	0.54 ± 0.04 **BC**	0.56 ± 0.03 **B**	0.51 ± 0.03 **C**
**Juice pH**	3.56 ± 0.05 **A**	3.51 ± 0.07 **A**	3.56 ± 0.03 **A**	3.65 ± 0.03 **A**	3.60 ± 0.01 **A**	3.56 ± 0.02 **A**	3.61 ± 0.05 **A**	3.61 ± 0.04 **A**
**Berry weight (g)**	6.66 ± 1.40 **A**	7.42 ± 0.20 **A**	7.86 ± 0.58 **A**	7.36 ± 0.55 **A**	7.16 ± 0.59 **A**	7.23 ± 0.14 **A**	8.14 ± 0.97 **A**	7.26 ± 0.42 **A**
**Berry length (mm)**	27.52 ± 1.14 **A**	27.71 ± 0.65**A**	27.84 ± 0.97 **A**	27.13 ± 0.27 **A**	27.67 ± 1.04 **A**	27.49 ± 0.26 **A**	28.65 ± 1.79 **A**	27.38 ± 0.67 **A**
**Berry width (mm)**	20.52 ± 0.63 **A**	20.74 ± 0.20 A	21.08 ± 0.59 **A**	20.31 ± 0.10 **A**	20.63 ± 0.38 **A**	20.59 ± 0.15 **A**	20.94 ± 0.89 **A**	20.59 ± 0.45 **A**
