# Supplementary material for: The Complexity of Modulating Anthocyanin Biosynthesis Pathway by Deficit Irrigation in Table Grapes
Source: Front Plant Sci. 2021 Aug 18;12:713277. doi: 10.3389/fpls.2021.713277 (PMC8416356; doi:10.3389/fpls.2021.713277)
Supplement: Supplementary Table 2 — Primers for genes used for RT-qPCR. [file Data_Sheet_6.PDF]

Supplementary Table 2. Primers for genes used for RT-qPCR.

Gene	Code	Sequence	Forward primer	Reverse primer
Flavanone 3-hydroxylase	*F3H*	X75965	CCAATCATAGCAGACTGTCC	TCAGAGGATACACGGTTGCC
Chalcone synthase	*CHS2*	AB066275	GAAGATGGGAATGGCTGCTG	AAGGCACAGGGACACAAAAG
VvmybA1 transcription factor	*MybA1*	AB097923	TAGTCACCACTTCAAAAAGG	GAATGTGTTTGGGGTTTATC
UDP-glucose: flavonoid-3-O-glucosyltransferase	*UFGT*	AF000372	GGGATGGTAATGGCTGTGG	ACATGGGTGGAGAGTGAGTT
9-cis-epoxycarotenoid dioxygenase	*VvNCED1*	AY337613	TTTGTGCACGACGAGAAGAC	TCTGCAATCTGACACCAAGC
Superoxide dismutase1	*SOD1*	GSVIVT00029451001	GGCGATTCATCTACGGTTGT	CAACCCAGTGAACCTTTTGG
Superoxide dismutase3	*SOD3*	GSVIVT00008877001	AATGAGGGTGCTTGTGGAAC	AGGCCAGAAAGACTCCCAGT
Ascorbate peroxidase1	*ASPX1*	GSVIVT00024455001	AAATGGGTCTCAGCGACAAG	CAGGGTCCTTCAAATCCAGA
Ascorbate peroxidase3	*ASPX3*	GSVIVT00015409001	GAAATTTGTGGCAGCCAAGT	GCTTCCACCAACTGCTTCAT
GA 2-oxidase1	*GA2OX1*	KC898179	TGCCAACTCCTTCTTCATCA	CCAAAACCCTATGCCTCACA
ACC oxidase3	*ACO3*	359485521	CCGAGCCCACACTGATGCCG	TGGAGTGGCGCATTGGAGGA
VvMYCA1	*MYCA1*	EF193002	GAACAGGAGGGGATGAGTGA	CTTGGGAAGCACCTCCATTA
VvWDR1	*WDR1*	DQ517913	GCATTCTGAGGGAGATGGTC	TCCGAATCAAGAACCAAAGC
Ubiquitin	*UbiC*	TC38636	TCTGAG GCTTCGTGGTGGTA	AGGCG TGCATAACATTTGCG
